# Drug Reaction with Eosinophilia and Systemic Symptoms: DRESS following Initiation of Oxcarbazepine with Elevated Human Herpesvirus-6 Titer

**DOI:** 10.1155/2014/853281

**Published:** 2014-02-12

**Authors:** Seth L. Cornell, Daniel DiBlasi, Navin S. Arora

**Affiliations:** ^1^Department of Medicine, Tripler Army Medical Center, Honolulu, HI 96859, USA; ^2^Dermatology Service, Tripler Army Medical Center, Honolulu, HI 96859, USA

## Abstract

Drug reaction with eosinophilia and systemic symptoms (DRESS) is a rare and potentially fatal severe cutaneous reaction, which has a delayed onset after the initiation of an inciting medication. After recognition and withdrawal of the causative agent, along with aggressive management, a majority of patients will have complete recovery over several months. We present a rare case of DRESS secondary to oxcarbazepine with an elevated human herpesvirus-6 titer.

## 1. Introduction

Drug reaction with eosinophilia and systemic symptoms (DRESS) is a rare, severe, cutaneous reaction that was prototypically associated with aromatic anticonvulsant medications; however, it is now recognized that it can be caused by a variety of pharmacologic agents. Although no consensus has been reached regarding its pathogenesis, reactivation of human herpesvirus-6 (HHV-6) has been associated with DRESS. Presentation typically occurs within six to eight weeks after initiation of an offending medication and often resolves with prompt discontinuation; however, fatal cases have been reported. Here, we present a rare case of DRESS secondary to oxcarbazepine and associated with elevated HHV-6 titer.

## 2. Case Report

A 29-year-old Asian female presented to the emergency department for a progressively worsening rash over the prior week. The eruption originated as a solitary pruritic plaque on her left arm, which over the next two days spread to her trunk and legs. Her family physician initially prescribed a course of valacyclovir for presumed varicella zoster virus infection. She returned to the same provider three days later and the rash was noted to now involve the interdigital aspects of her hands and feet. Permethrin cream was prescribed due to concern for scabies. The lesions continued to worsen and the patient developed a fever and sore throat eight days after the eruption onset. She was subsequently instructed by her primary care provider to go to the emergency department for further evaluation.

**Figure 1:**
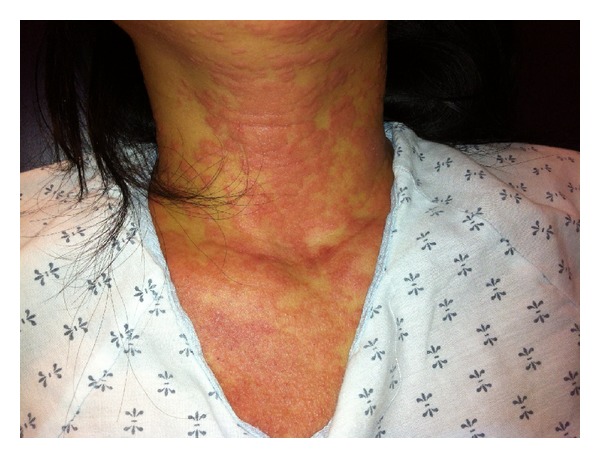
Anterior neck and upper chest: discrete and coalescing, slightly blanching, planar plaques.

**Figure 2:**
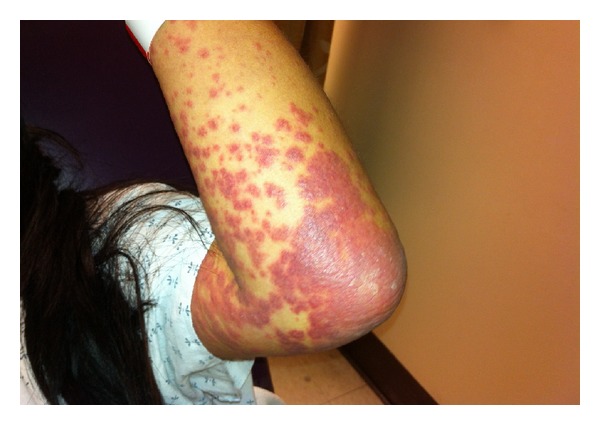
Left ventral arm/elbow: discrete and coalescing, nonblanching papules coalescing into plaques, most consistent with a palpable purpura.

**Figure 3:**
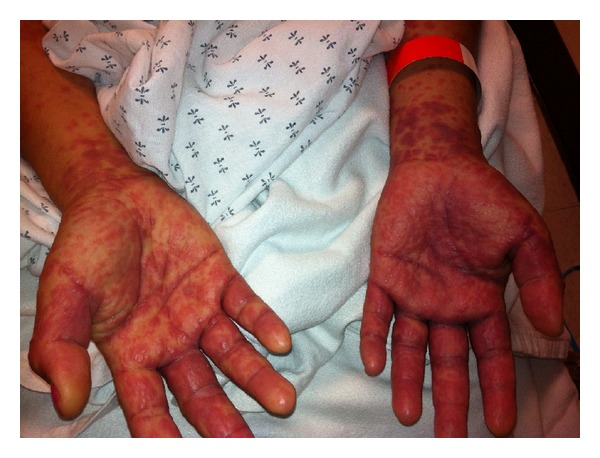
Bilateral wrists/palms: tender, nonblanching red violaceous papules and plaques, some with early vesiculation.

In the emergency department, she complained of severe pruritis and painful oral lesions but denied having any painful skin lesions, skin sloughing, or any anogenital lesions. On exam, she was tachycardic to 131 bpm but was afebrile, normotensive, and in no acute distress. Cardiovascular and pulmonary examinations were unremarkable. Cutaneous examination revealed numerous erythematous discrete papules and minimal blanching on the bilaterally distal extremities, face, and neck (Figure 1). There were also discrete papules coalescing into nonblanching, erythematous plaques on her trunk and proximal extremities (Figures 2 and 3). The soft palate did exhibit petechiae, without any erosions or ulcerations. Cervical lymphadenopathy was also appreciated.

The patient had a history of Hodgkin lymphoma diagnosed, in 2007, at the age of 25, which was treated with chemotherapy and electron beam radiotherapy, and has been in remission since then. Medications included venlafaxine for depression and oxcarbazepine for mood stabilization which was started approximately 2 months before. She endorsed allergies to latex and shellfish and admitted smoking less than one pack of cigarettes per day.

A cell blood count was notable for atypical lymphocytes at 9% (1.1 × 10^9^/L) and elevated eosinophils of 7% (0.68 × 10^9^/L). Liver enzymes were also elevated, with alanine aminotransferase at 130 units/L (15–46 units/L), aspartate aminotransferase at 108 units/L (13–69 units/L), and alkaline phosphatase of 267 units/L (38–126 units/L). Chemistry and coagulation studies were unremarkable. Despite a thorough discussion regarding the necessity of a skin biopsy, the patient declined.

The patient was diagnosed with DRESS based on a “definite” RegiSCAR score and subsequently admitted to the hospital. Oxcarbazepine was discontinued and prednisone was initiated at 1.5 mg/kg daily. During the hospitalization, liver enzymes downtrended, while the eosinophilia increased from 7% to 12% (1.55 × 10^9^/L). An HHV-6 IgG level was checked 1 week after admission and was elevated at 7.96 IV (>1.11 indicates current or past infection).

The patient was discharged on hospital day three and was continued on prednisone 1.5 mg/kg daily, given the stabilization of her eruption. She was educated on the importance of avoiding aromatic epileptic drugs and to follow up with her psychiatrist for medication reevaluation.

One week after discharge, her eruption continued to improve and her lymphadenopathy had resolved. The lesions became blanchable on the trunk; however, her eosinophilia increased from 12% to 23% (4.68 × 10^9^/L). On postdischarge day fifteen, there was a decrease in her edema with truncal desquamation. Prednisone dose was decreased to 1 mg/kg. On postdischarge day 27, the lesions had almost completely resolved, with residual postinflammatory hyperpigmentation observed on bilateral ankles. The prednisone was tapered off over the next month with total resolution of the rash and normalization of eosinophils (0.23 × 10^9^/L).

## 3. Discussion

Adverse cutaneous reactions occur in approximately two to three percent of hospitalized patients [1]. Severe cutaneous reactions such as toxic epidermal necrolysis, Steven-Johnson Syndrome, angioedema, and serum sickness have been estimated to occur in about 1 out of every 1000 patients hospitalized [2].

The term DRESS was first proposed in 1996, though medical providers had likely been encountering this severe cutaneous adverse reaction (SCAR) since the advent of hydantoin derivatives for the treatment of convulsive disorders [3, 4]. First described in 1959, as a pseudolymphoma often accompanied by exanthem, eosinophilia, and fever, this potentially fatal SCAR has been known by a variety of names including drug-induced hypersensitivity syndrome, drug-induced delayed multiorgan hypersensitivity syndrome, anticonvulsant hypersensitivity syndrome, and phenytoin syndrome [5]. The lack of a standard nomenclature has caused considerable diagnostic confusion.

Originally described in 1996, the proposed diagnosis of DRESS required a cutaneous drug eruption with both hematologic abnormalities (eosinophilia or atypical lymphocytes) and systemic involvement (adenopathy, hepatitis, nephritis, pneumonitis, or carditis). More recently, a diagnostic scoring system has been proposed by the European Registry of Severe Cutaneous Adverse Reactions (RegiSCAR) that helps clinicians determine if DRESS is definite, probable, possible, or excluded [6]. According to this classification system, features consistent with DRESS include eosinophilia, fever, lymphadenopathy, atypical lymphocytes, leukopenia, diffuse rash, organ involvement, and disease duration greater than fifteen days. Determining the incidence of DRESS has been historically difficult given its discordant diagnostic denotation, though it has been estimated at between 1 in 1,000 and 1 in 10,000 drug exposures [7].

Although the exact pathogenesis of DRESS is not completely understood, proposed mechanisms include reactive drug metabolite formation with subsequent immunologic activation, slow acetylation, and reactivation of HHV-6 [8, 9]. DRESS has been strongly associated with aromatic anticonvulsant agents such as phenytoin, phenobarbital, and carbamazepine; however, several other classes of medications are also associated with DRESS.

The mainstay of treatment of DRESS is prompt diagnosis and removal of the offending agent. Corticosteroids are routinely used, though consensus regarding the dose and route of administration has not been established. Treatment with intravenous immune globulin has also been described [10]. After diagnosis, recovery typically occurs within six to nine weeks; however, mortality has been estimated at approximately 5% [11]. DRESS may also predispose affected patients to long-term autoimmune sequelae, most notably thyroid dysfunction, along with type 1 diabetes mellitus, and autoimmune hemolytic anemia [12].

Our case demonstrates typical symptoms of DRESS, such as the delayed onset of cutaneous lesions after the initiation of an aromatic anticonvulsant, peripheral eosinophilia, hepatitis, lymphadenopathy, and prolonged disease duration. The patient's symptoms slowly resolved with the removal of oxcarbazepine and the initiation of oral corticosteroid therapy. This case highlights a rarely described cause of DRESS with both oxcarbazepine and an elevated HHV-6 antibody likely implicated in disease development.

## 4. Conclusion

DRESS must be considered in all patients presenting with a cutaneous eruption and visceral organ involvement, with recent initiation of a new medication, especially aromatic anticonvulsants. The delayed onset of symptoms following initiation of a new medication and an elevated HHV-6 titer are suggestive of DRESS and may help differentiate it from other forms of severe cutaneous adverse reactions. Prompt recognition of DRESS, with immediate cessation of the offending medication, is paramount in treating this potentially fatal disease process.
